# The cullin4A is up-regulated in chronic obstructive pulmonary disease patient and contributes to epithelial-mesenchymal transition in small airway epithelium

**DOI:** 10.1186/s12931-019-1048-4

**Published:** 2019-05-06

**Authors:** Yidan Ren, Yi Zhang, Lixia Fan, Qinlian Jiao, Yunshan Wang, Qin Wang

**Affiliations:** 1 Department of Anesthesiology, Qilu Hospital, Shandong University, Jinan, China; 2 Department of Respiratory Medicine, Qilu Hospital, Shandong University, Jinan, China; 3 Department of Clinical Laboratory, The Second Hospital of Shandong University, Jinan, China; 4 International Biotechnology R&D Center, Shandong University School of Ocean, Weihai, China

**Keywords:** CUL4A, Chronic obstructive pulmonary disease, Small airway epithelial cells, Epithelial-mesenchymal transition, Cigarette smoke

## Abstract

**Background:**

Chronic obstructive pulmonary disease (COPD) is a common respiratory disease with high morbidity and mortality. The most important pathophysiological change of COPD is airway obstruction. Airway obstruction can cause airflow restriction and obstructive ventilation dysfunction. Currently, many studies have shown that there is EMT phenomenon in the process of airway remodeling of COPD. Cullin4A (CUL4A) is an E3 ubiquitin ligase that interacts with other factors to form the E3 complex. Studies have shown that CLU4A is associated with EMT in non-small cell lung cancer and other cancers. However, its relationship with EMT in COPD has not been reported systematically. In this study, we detected the expression of CUL4A in lung epithelium of COPD patients. In addition, the regulatory effect and mechanism of CUL4A on EMT in COPD were clarified in small airway epithelial cells.

**Methods:**

The expression of CUL4A was assessed by immunohistochemistry in lung epithelium specimens from smokers, non-smokers and patients with chronic obstructive pulmonary disease. The role of CUL4A on cigarette smoke extract (CSE)-induced epithelial-mesenchymal transition (EMT) in human small airway epithelial cells (HSAEpiCs) was assessed by silencing or overexpression CUL4A in vitro. Cigarette smoke is recognized as a high-risk factor in the induction of COPD, and its damage to the airway involves airway damage, airway inflammation and airway remodeling.

**Results:**

The results shown that CUL4A expression in small airway epithelium was significantly increased in patients with COPD. We also observed a significant negative association between CUL4A and FEV_1_%, a useful clinical marker for the diagnosis and evaluation of COPD severity, in small airway epithelial cells. In vitro, CSE-induced EMT is associated with high expression of CUL4A, and targeted silencing of CUL4A with shRNA inhibits CSE-induced EMT in human small airway epithelial cells.

**Conclusions:**

Our results showed that CUL4A was overexpressed in lung epithelium of COPD patients, and CUL4A could regulate EMT of human small airway epithelium, which revealed a new mechanism of remodeling of small airway epithelium of COPD patients.

**Electronic supplementary material:**

The online version of this article (10.1186/s12931-019-1048-4) contains supplementary material, which is available to authorized users.

## Background

Chronic obstructive pulmonary disease (COPD) is a common respiratory disease. As its morbidity and mortality increase, it has become an important public health problem [1]. Despite the increasing research on COPD in recent years, the understanding of the pathogenesis of COPD is far from enough, and there is still no effective prevention and treatment [2, 3]. COPD is characterized by small airway wall thickening, which damages the integrity of lung tissue and leads to incomplete reversible airflow limitation, resulting in a series of clinical manifestation [4]. Clinicopathological studies have shown that small airway epithelial epithelial-mesenchymal transformation (EMT) is one of the important initial factors for COPD small airway wall thickening and fibrosis [5]. Studies have shown that excessive accumulation of myofibroblasts around the small airways is the main cause of small airway wall thickening, and EMT is one of the main sources of myofibroblasts around the small airways [6]. Therefore, in-depth study of the mechanism of EMT in COPD small airway epithelial cells and its influencing factors is of great significance to the elucidation of the pathogenesis of COPD.

EMT is a biological phenomenon in which epithelial cells are transformed into mesenchymal cells through specific processes. It plays an important role in embryonic development, chronic inflammation, tissue remodeling, tumor metastasis, and various fibrosis diseases [7]. The main characteristics of EMT include the decrease of cell adhesion molecules expression, the transformation of cytokeratin skeleton into vimentin-based skeleton, and the morphological characteristics of mesenchymal cells [8]. Numerous studies have confirmed that EMT is closely related to the occurrence and development of fibrosis in various tissue and organ [9]. Our previous studies on COPD also confirmed the existence of EMT in small airway epithelial cells of COPD, and found that uPAR system plays an important regulatory role in EMT of small airway epithelial cells [10].

Ubiquitin is a polypeptide composed of 76 amino acid residues, which participate in protein degradation [11]. E3 ubiquitin ligase plays a key role in recognition of substrate, which determines the specificity of the reaction [12]. CUL4A is a E3 ubiquitin ligase that interacts with other factors to form the E3 complex. Studies have shown that CUL4A is overexpressed in a variety of tumors [13–18]. Our previous study found that CUL4A is overexpressed in non-small cell lung cancer tissues and can promote the proliferation of lung cancer cells [19]. In addition, our study also found that CUL4A can regulate EMT of tumor cells [19, 20]. However, whether CUL4A is involved in the development of COPD and can regulate EMT in small airway epithelial cells in COPD patients has not been reported. Based on the close relationship between COPD and lung cancer, we speculate that CUL4A may also plays an important role in the development of COPD.

In this study, we observed the overexpression of CUL4A in COPD patients by immunohistochemical analysis. In vitro experiments confirmed that CUL4A plays an important role in regulating EMT in human small airway epithelial cells (HSAEpiCs), and revealed its possible molecular mechanism. The significance of this study is not only to systematically analyze the biological function and clinical significance of CUl4A in COPD, but also to reveal the possible mechanism of small airway cell remodeling in COPD.

## Methods

### Chemicals and antibodies

The TRizol Reagent (# 10296010) and Lipofectamine 3000 transfection (# L3000001) was purchased from Invitrogen, the reverse transcription kit (# RR037A) and the fluorescent quantitative PCR kit (# RR420A) were purchased from TAKARA. The immunohistochemistry kit was purchased from ZhongShan Biotech(SP-9000). CUL4A antibody was purchased from Sigma (# SAB1406671), Antibodies against E-cadherin, N-cadherin, vimentin, alpha-catenin, and Slug were purchased from Cell Signaling Technology (# 9782). All other chemicals were from Sigma unless otherwise stated.

### Patient

The lung tissue samples required in the experiment were obtained from 55 patients (17 non-smokers = never smoker group; 15 former smokers without COPD = former smoker group; 23 smoke patients with COPD = COPD group) who underwent lobectomy or pneumonectomy in Qilu Hospital (Jinan, China) (Table 1), and the patient recruited for this study is a retrospective study. Matched control never-smokers and control ex-smokers were enrolled. Ex-smokers had smoking history of ≥10 pack-years and had quit smoking for at least 1 year. Current smokers and patients with asthma were excluded. The Global Initiative Guide to Obstructive Pulmonary Disease is used to diagnose COPD. Subjects did not take corticosteroids (oral or inhaled) prior to tissue collection. All the experiments were approved by the ethics committee of Qilu Hospital. Before collecting specimens, the informed consent of all patients was obtained.

**Table 1:** Demographic characteristics of the subjects

	Control never-smoker (CNS) *n* = 23	Control ex-smoker (CES) *n* = 15	COPD *n* = 17
Sex (female/male)	16/7	2/13	1/16
Age (years)	52 ± 5	55 ± 9	62 ± 6
Smoking history, pack-years	–	19 ± 13	35 ± 17
FEV1, % predicted	93 ± 10	96 ± 12	68 ± 17
FEV1/FVC %	87 ± 3	82 ± 8	59 ± 6
GOLD stage			
1	–	–	2
2	–	–	10
3	–	–	5
4	–	–	–

Values are given as mean ± SD

Pack-year = one-year smoking 20 cigarettes per day

*COPD* chronic obstructive pulmonary disease, *FEV1* forced expiratory volume in 1 s, *FVC* forced vital capacity

### Immunohistochemistry

The obtained lung tissue was fixed with formalin, embedded in paraffin and sectioned for immunohistochemical staining. The specific steps are as follows: the tissue of the paraffin section is subjected to antigen retrieval, and then the activity of endogenous peroxidase is blocked by hydrogen peroxide; blocking with serum, and then incubating the tissue section with the primary antibody recognizing CUL4A at 4 °C overnight; PBS wash three times, add the corresponding secondary antibody, 30 min at room temperature, wash PBS three times, add the third antibody, incubate for 15 min at room temperature; wash three times with PBS, DAB treating, hematoxylin staining, hydrochloric acid alcohol differentiation, dehydration, sealing, microscopic observation of staining. Photographs were taken using an OLYMPUS IX81 optical microscope (Olympus, Tokyo, Japan) equipped with a SPOT camera. Image analysis was performed using Image-Pro Plus 6.0 software (Media Cybernetics, Silver Spring, MD, USA). The area of the small airway epithelia and length of the basement membrane were evaluated. CUL4A was expressed as the number of positive epithelial cells/mm basement membrane. All slides were analyzed in a single batch by a single experienced observer with quality assurance on randomly selected slides provided by a professional academic pathologist.

### Cell culture

Human small airway epithelial cells (HSAEpiCs) were purchased from ScienCell Research Laboratories (catalog number 3231). The cells were cultured using small airway epithelial cell culture medium (SAEpiCM) and cultured at 37 °C, 5% carbon dioxide. In the EMT studies, the number of passage times of cells was less than 10 generations.

### Preparation of CSE

The preparation of CSE is based on the methods used in previous laboratory studies. In short, a non-filtered commercial cigarette (containing 13 mg tar and 1.2 mg nicotine per cigarette) was burned with a special syringe drive device, and mainstream smoke was fed into serum-free F12 medium of 20 ml. The pH was adjusted to 7.4, and the bacteria were removed by 0.22 um pore filter. The solution (designed as 100% CSE solution) will be used within 30 min after preparation.

### Construction of CUL4A overexpressing and cell transfection

HSAEpiC cells that overexpress human CUL4A were prepared by transfecting cells with pWZL-CUL4A or with empty vector using Lipofectamine 3000 (Invitrogen, Carlsbad, CA, USA) in accordance with the manufacturer’s instructions. Cells were trypsinized and subjected to various experiments. The expression of CUL4A was confirmed by qRT-PCR and Western blot.

### CUL4A specific short hairpin RNA suppression

To knock out CUL4A expression in HSAEpiCs, short hairpin RNA (shRNA) targeting human CUL4A was cloned into the pSuper vector, and a control oligonucleotide sequence corresponding to the inverse CUL4A shRNA sequences was prepared. When the cells were grown to about 75%, transfection was performed with pSuper-shRNA targeting CUL4A or empty vector using Lipofectamine 3000 (Invitrogen, Carlsbad, CA, USA) according to the manufacturer’s instructions. After 24 h, the cells were digested and used in various experiments. The knockdown effect of CUL4A was assessed by qRT-PCR and Western blot.

### qRT-PCR

Total RNA was extracted using Trizol reagent (Invitrogen), the mass and concentration of the obtained RNA was determined by ultraviolet spectrophotometer. Reverse transcription of total RNA (1 μg) into cDNA (20 μl) using the PrimeScriptTM RT Kit (TaKaRa) according to the manufacturer’s instructions。PCR reaction with 1 μL of cDNA product, 0.3 μL of forward primer (10 μmol/L), 0.3 μL of reverse primer (10 μmol/L), 0.2 μL of ROX Reference Dye II, 5 μLTB green, 3.5 RNase-free water (TaKaRa). Endogenous GAPDH was used as a standardized control. Relative quantification of mRNA was performed by comparing CT values.

### Western blot

Total cell protein was extracted using RIPA containing a complete protease inhibitor, separated by 10% SDS-PAGE, and transferred to PVDF membrane by wet transfer method to detect CUL4A, E-cadherin, α-catenin, N-cadherin and Vimentin. The expression was visualized using the ECL Plus system to capture images.

### MTT assay

The cells were made into cell suspensions and plated in 96-well plates at approximately 1000 cells per well. The tests were carried out 24, 48, 72, 96 h after plating. Added 20 μl of 5 mg/ml MTT solution to each well, and the culture medium was discarded after incubation at 37 °C for 4 to 6 h, 150 μl of DMSO was added to each well. Spectrophotometry was performed at a wavelength of 570 nm.

### Cell proliferation assay

The cells were seeded in a 96-well plate and incubated overnight. Cell proliferation was measured using a Cell Proliferation BrdU ELISA kit (Roche Diagnostics Ltd., Burgess Hill, West Sussex, UK) according to manufacturer’s instructions. Ultimately, the absorbance was recorded at 450 nm using a microplate reader (Bio-Rad Laboratories Inc., Hercules, CA, USA). The experiment was performed in triplicate.

### Cell invasion and motility assay

Invasion of cells was measured in Matrigel (BD, Franklin Lakes, NJ, USA) -coated Transwell inserts (6.5 mm, Costar, Manassas, VA, USA) containing polycarbonate filters with 8-μm pores. According to the manufacturer’s recommendations, the inserts were coated with 50 μl of 1 mg/ml Matrigel matrix. 2 × 10^5^ Cells were plated in the upper chamber with 200 μl of serum-free medium, while 600 μl of medium with 10% fatal bovine serum were added to lower well. Top cells were removed and bottom cells were counted after 24 h’ incubation. Cells were fixed in 4% paraformaldehyde and stained with 0.5% crystal violet after migrating to the lower surface of the membrane. Five random fields were counted at × 10 magnification for each membrane. The mean was calculated from three independent experiments done in triplicate. Motility assays were similar to Matrigel invasion assay except that the Transwell insert was not coated with Matrigel.

### Immunofluorescence

The cells were prepared as cell suspensions, plated in a 24-well plate with cell slide at a density of about 5000 per well, cultured at 37 °C, 5% CO _2_ for appropriate time. Discarded in the medium; fixed with PFA, punched with 0.4% Triton-100; and blocked by horse serum; incubated with the corresponding primary antibody at 4 °C overnight; PBS washed for 3 times; and the fluorescent secondary antibody was incubated for 1 h at room temperature; washed again, and finally the cells were stained with DAPI. Laser confocal microscopy was used to take pictures and collect pictures.

### Chromatin immunoprecipitation-quantitative PCR (ChIP-qPCR)

The chromatin immunoprecipitation (ChIP) Kit was purchased from Millipore and ChIP experiments were carried out essentially as described previously [19]. Immnuoprecipitated DNA was analyzed on the ABI PRISM 7900HT sequence detection system. The primers used for detection of promoters after ChIP are available upon request.

### Statistical analysis

Statistical analysis was performed using the SPSS statistical software program (IBM. Armonk, New York, USA). Data are expressed as mean ± SD. The association between CUL4A and SLUG in lung tissue samples was assessed by X^2^ test. Comparisons between the different groups were performed using Student’s t-test. The dominant setting was *p* < 0.05.

## Results

### CUL4A is up-regulated in human COPD small airway epithelial cells and correlated with the forced expiratory volume of predicted (FEV_1_%)

**Fig. 1:**
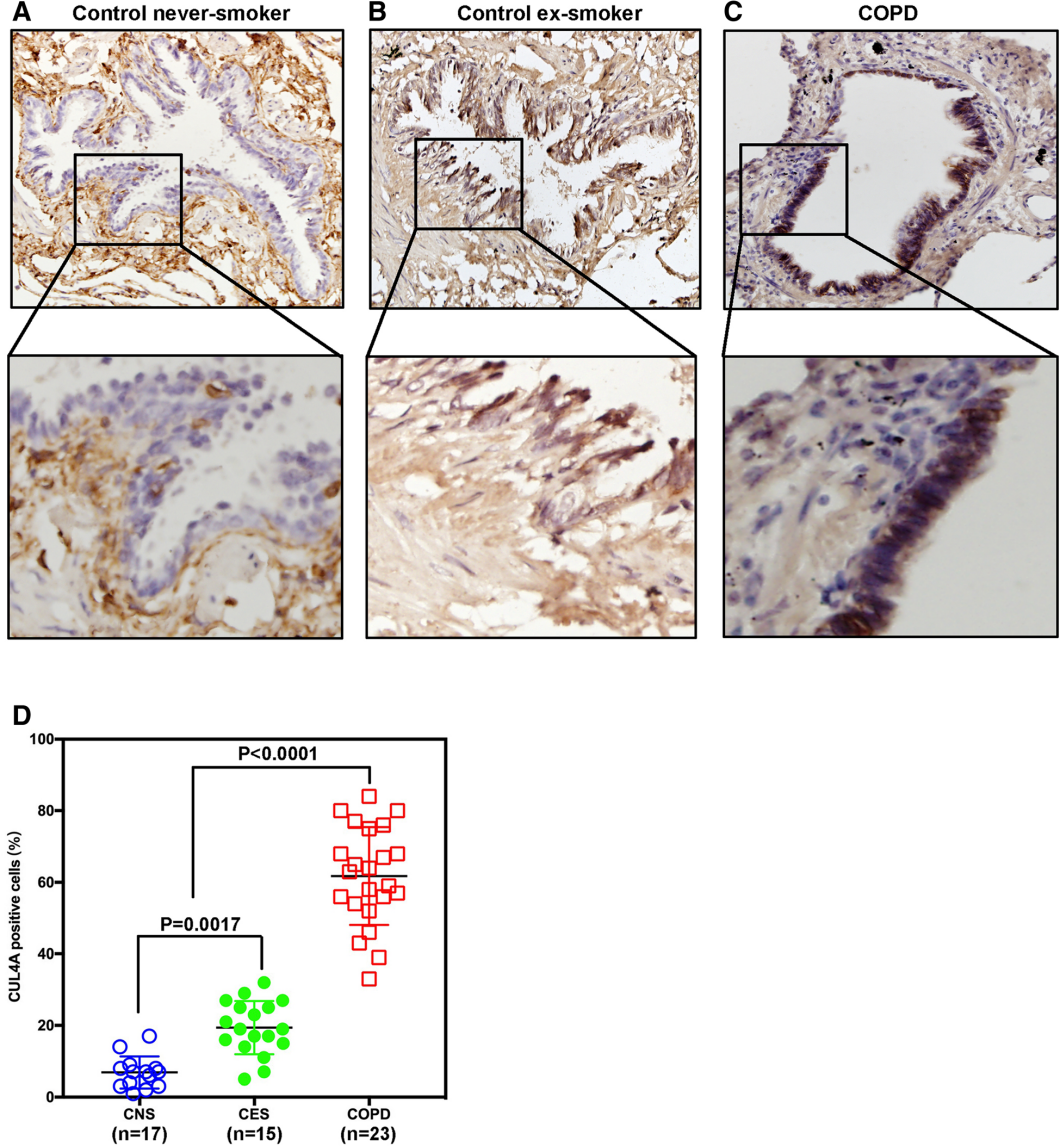
Immunohistochemistry of CUL4A in small airway epithelium. Representative photographs of the immunoreactivity against CUL4A in human small airways are shown. **a** Immunohistochemical staining of CUL4A in small airway epithelium from control never smokers; **b** CUL4A expression in small airway epithelium in smokers without COPD; **c** CUL4A expression in small airway epithelium in patients with COPD. The upper panels show low-magnification images, and the lower panels show magnified images; **d** Quantitative analysis of CUL4A positive cells in small airway epithelium. CNS, control never-smoker; CES, control ex-smoker; COPD, ex-smoker with COPD

**Fig. 2:**
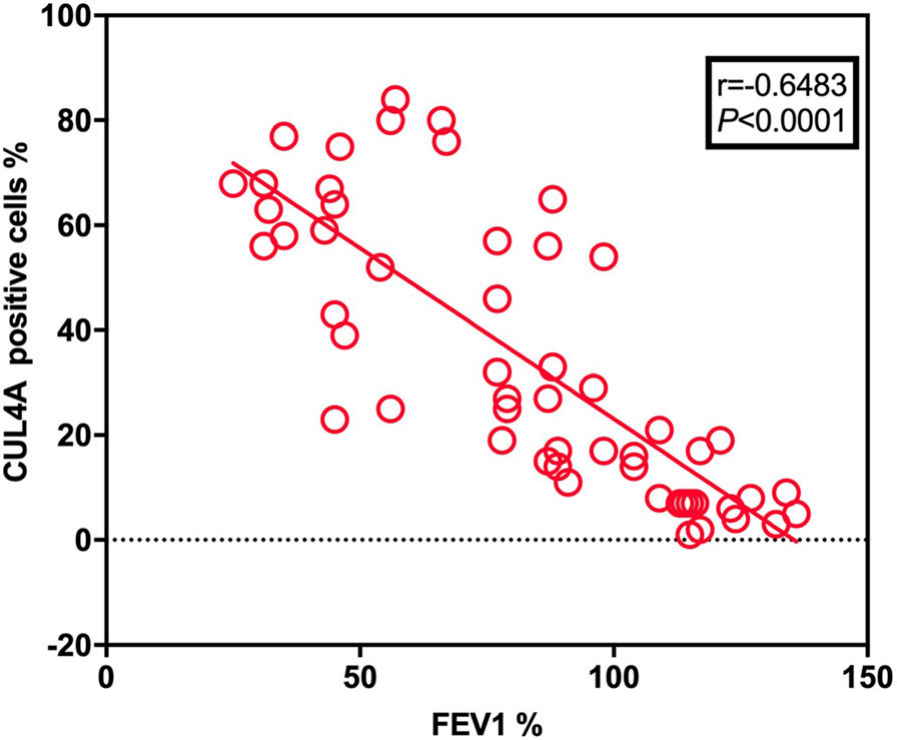
Correlations between the levels of CLU4A and the values of FEV_1_% predicted

To investigate whether CUL4A is highly expressed in patients with COPD. First, we performed immunohistochemical staining on a number of lung tissue sections from COPD patients and their control group (Fig. 1a, b and c). Positive staining was calculated using image-pro + 6.0 to evaluate CUL4A expression. No significant or weak CUL4A immune response was observed in the control group. CUL4A expression in lung epithelium was significantly higher in smokers and COPD patients than in nonsmokers (Fig. 1d). Predictive forced expiratory volume (FEV_1_%) was used for the diagnosis and severity assessment of COPD. The FEV_1_% of COPD patients was lower than that of normal people. In addition, we also found that the expression level of CLU4A was significantly negatively correlated with FEV_1_% (Fig. 2). The above results suggest that CUL4A has a significant correlation with COPD and may play a key regulatory role in the occurrence and development of COPD.

### CSE-induced CUL4A overexpression in cultured HSAEpiC cells

**Fig. 3:**
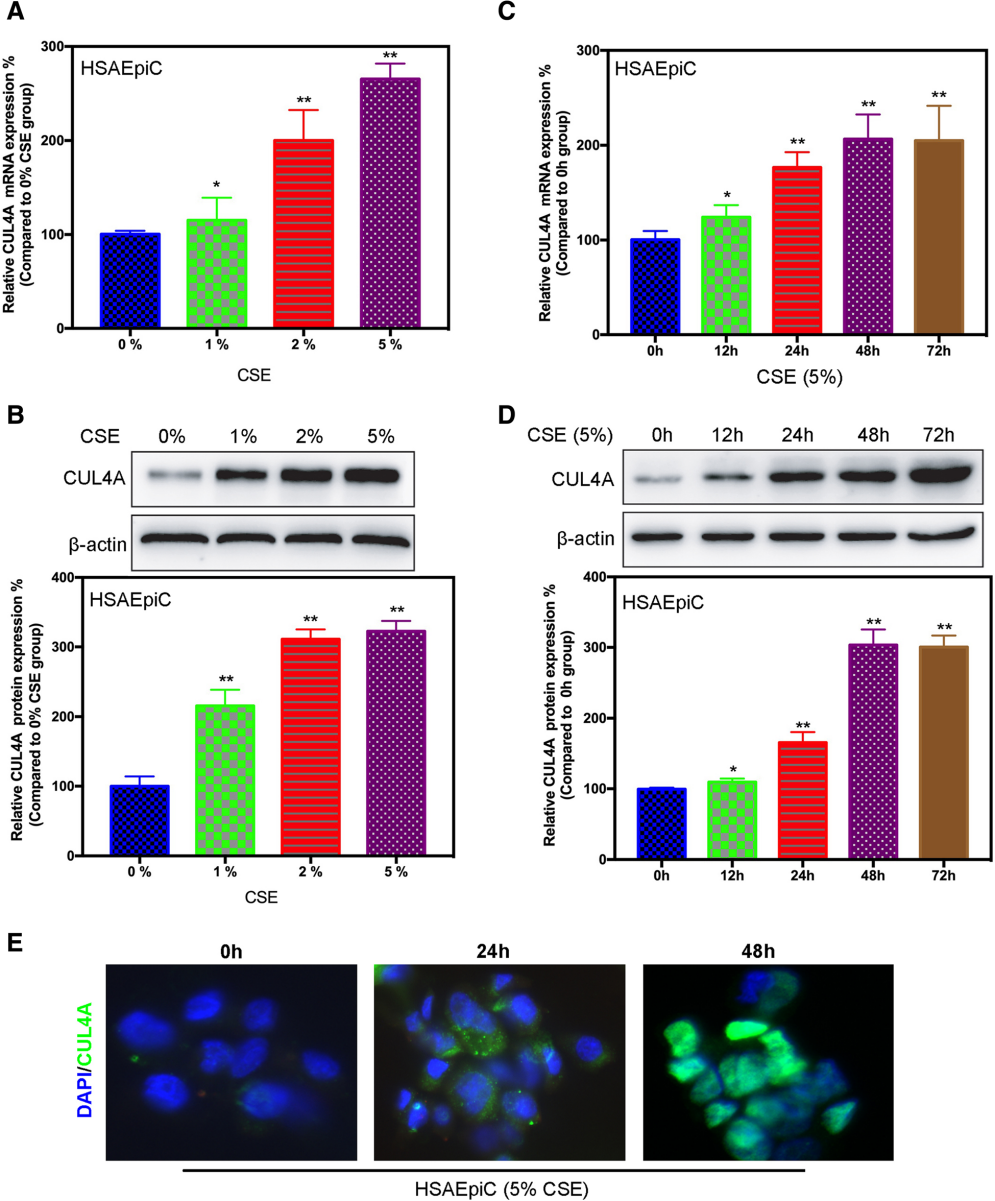
CSE promotes the expression of CUL4A in HSAEpiC cells. **a** qRT-PCR and **b** western blot analysis evaluation of CUL4A in HSAEpiC cells after 48 h of stimulation with CSE at 0, 1, 2, 5%. **c** qRT-PCR and **d** western blot analysis evaluation of CUL4A in HSAEpiC cells after 0, 12, 24, 48, 72 h of stimulation with CSE (5%). **e** Immunofluorescence stain evaluation of CUL4A in HSAEpiC cells after 0, 24, 48 h of stimulation with CSE (5%). *P < 0.05 and **P < 0.01 compared to 0 h. All results are from 3 independent experiments. Error bars indicate standard deviation

Smoking is the most common risk factor for COPD. The expression of CUL4A in lung epithelial cells was significantly elevated in smokers (Fig. 1b). Therefore, cigarette smoke extract (CSE) may can promote the expression of CUL4A. To better simulate this microenvironment, we cultured human small airway epithelial cells (HSAEpiCs) in the presence of CSE. We treated cells with different concentrations of CSE or the same concentration of CSE (5%) at different times, and measured the expression of CUL4A. The results show that CSE increases CUL4A mRNA expression in a concentration-dependent manner compared with untreated cells. CUL4A mRNA expression increases as CSE concentration increases (Fig. 3a). Western blot analysis confirmed the effect of CSE on CUL4A protein levels (Fig. 3b). Furthermore, we demonstrate that CSE increases CUL4A expression in a time-dependent manner. CUL4A expression increased with induction time (Fig. 3c-e).

### CUL4A regulates EMT in HSAEpiC cells

**Fig. 4:**
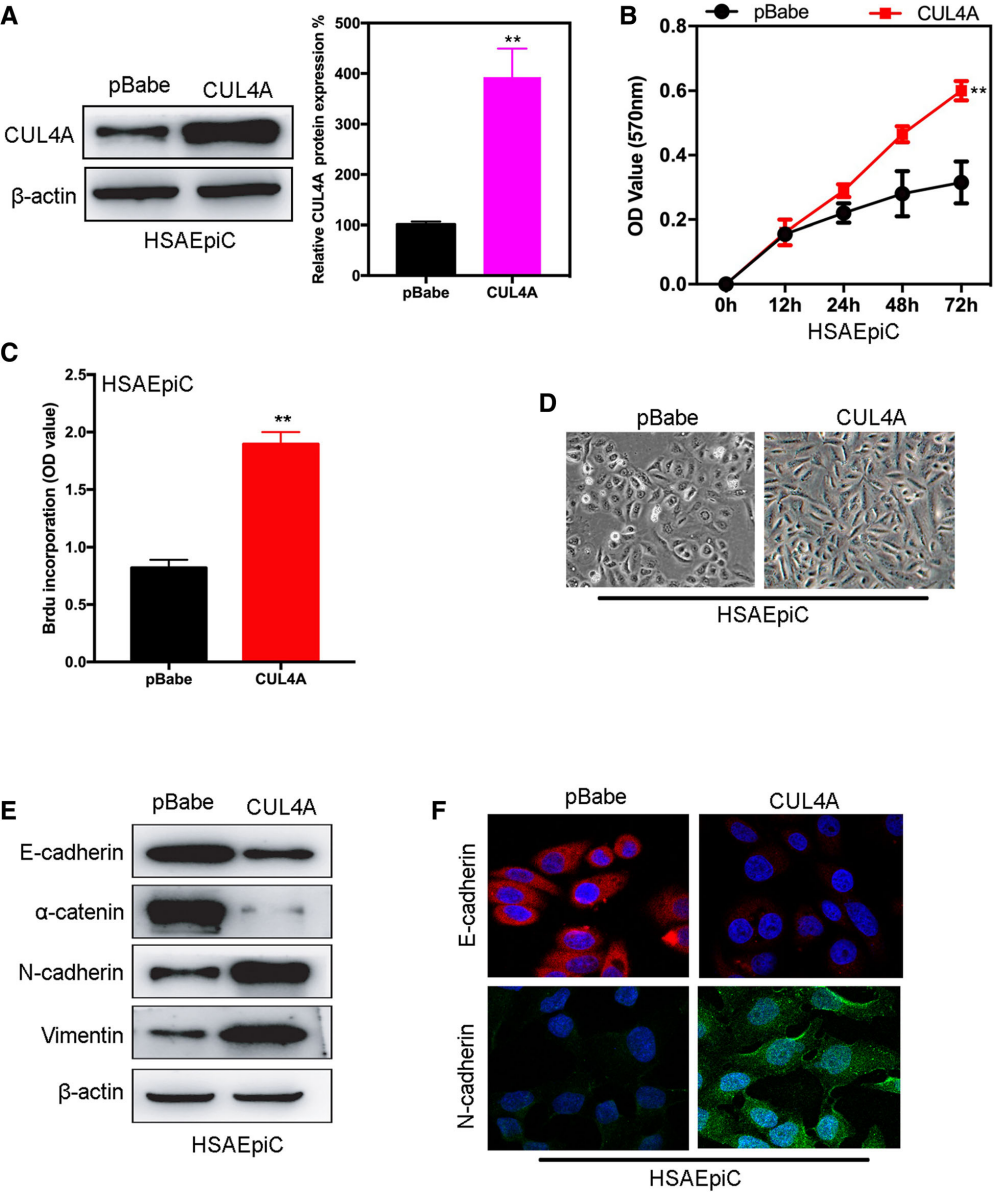
Epithelial-to-mesenchymal transition (EMT) in HSAEpiC cells was induced by CUL4A ectopic expression. **a** The expression of CUL4A was analysis by western blot in stable transfection of CUL4A HSAEpiC cells. **b** The proliferation of HSAEpiC cells after CUL4A overexpression was observed by MTT assay. **c** The proliferation of HSAEpiC cells after CUL4A overexpression was observed by BrdU-ELISA assay. **d** Changes in morphology of HSAEpiC-CUL4A cells were observed by microscopy. **e** After overexpression of CUL4A, the expression of epithelial (E-cadherin and α-catenin) and mesenchymal (N-cadherin and Vimentin) marker proteins was examined by Western blotting. **f** After overexpression of CUL4A, the expression of epithelial (E-cadherin) and mesenchymal (N-cadherin) protein markers was determined by immunofluorescence assay. **P < 0.01 compared to pBabe group. All results are from 3 independent experiments. Error bars indicate standard deviation

To establish a model for analyzing the functional role of CUL4A in regulating HSAEpiC cells, we used CUL4A or control vector (pBabe) to reverse transcriptional transfection of HSAEpiC cells and establish CUL4A overexpressed cell lines. Western blot and qRT-PCR confirmed increased expression of CUL4A in HSAEpiC-CUL4A cells compared with the control group (Fig. 4a). MTT and BrdU-ELISA assaies were used to detect the effect of CUL4A on HSAEpiC cell proliferation. HSAEpiC-CUL4A cells showed significantly increased proliferation compared to the empty vector control group (Fig. 4b and c). Our previous study found that CUL4A can regulate EMT of tumor cells. Therefore, CUL4A may also have the function of regulating EMT in HSAEpiC cell. In HSAEpiC-CUL4A cells, we observed changes in cell morphology through a microscope (Fig. 4d). Subsequently, several labeled epithelial and mesenchymal proteins were detected by Western blot and immunofluorescence assays. The results showed that HSAEpiCs cell lines overexpressing CUL4A showed significantly lower levels of cell surface E-cadherin and α-catenin expression compared with empty control cell lines, while N-cadherin and Vimentin expression levels were significantly increased (Fig. 4e and f). These results suggest that CUL4A induces EMT independently in HSAEpiC cells without exposure to CSE.

### Suppression of CUL4A attenuates CSE-induced EMT in HSAEpiC cells

**Fig. 5:**
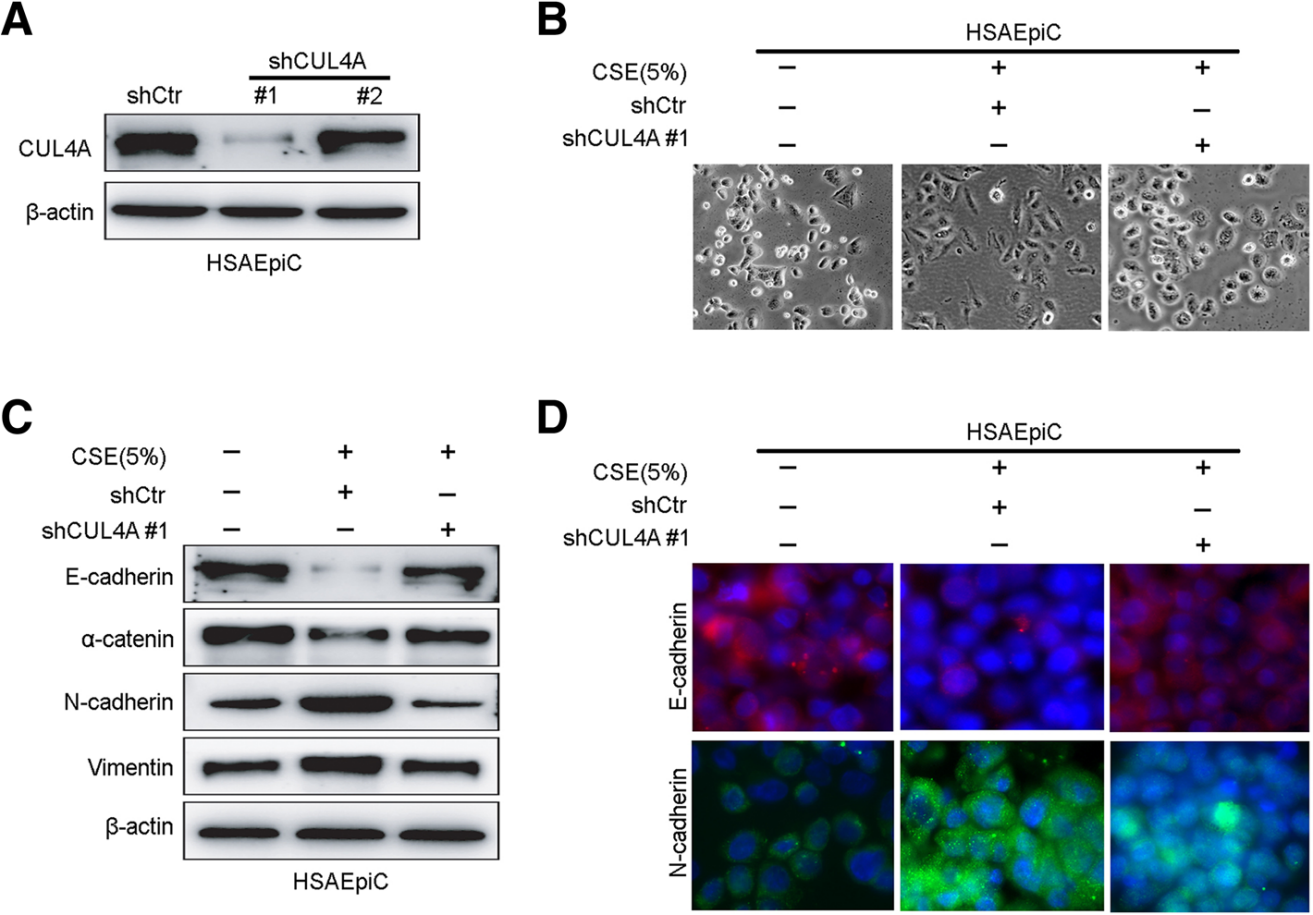
Suppression of CUL4A attenuates CSE-induced EMT in HSAEpiC cells. **a** Western blot was used to detect the interference efficiency of CUL4A. **b** Morphological changes of CSE-treated HSAEpiC cells after silencing CUL4A were observed. **c** Protein levels of E-cadherin, α-catenin, N-cadherin and vimentin were analyzed by western blot analysis in CSE-treated HSAEpiC cells after silencing CUL4A. **d** The expression of E-cadherin and N-cadherin protein markers was determined by immunofluorescence assay CSE-treated HSAEpiC cells after silencing CUL4A

As we mentioned before, CSE is considered to play an important role in HSAEpiC cell EMT. To further determine the specific biological function of CUL4A in CSE-induced EMT, we transfected HSAEpiC cells with shRNA expression plasmids targeting CUL4A to knock down CUL4A expression (Fig. 5E and F). BrdU-ELISA assay was used to detect the effect of CUL4A knocking down and CSE on HSAEpiC cell proliferation. HSAEpiC-shCUL4A cells showed significantly decreased proliferation compared to the control group (Additional file 1: Figure S1A). And CSE significantly increased proliferation of HSAEpiC cells (Additional file 1: Figure S1B). Next, we observed the morphological changes of CSE-stimulated HSAEpiC cells after silencing CUL4A expression. Compared with shControl Cells transfected with pSuper-shCUL4A show a more epithelial-like morphology (Fig. 5b), suggesting that interference with CUL4A significantly reverses CSE-induced EMT in HSAEpiC cells. Subsequently, Western blotting (Fig. 5c) and immunofluorescence assay (Fig. 5d) were used to detect the expression of multiple epithelial and mesenchymal marker proteins.

### CUL4A promotes the migration and invasion of HSAEpiC cells

**Fig. 6:**
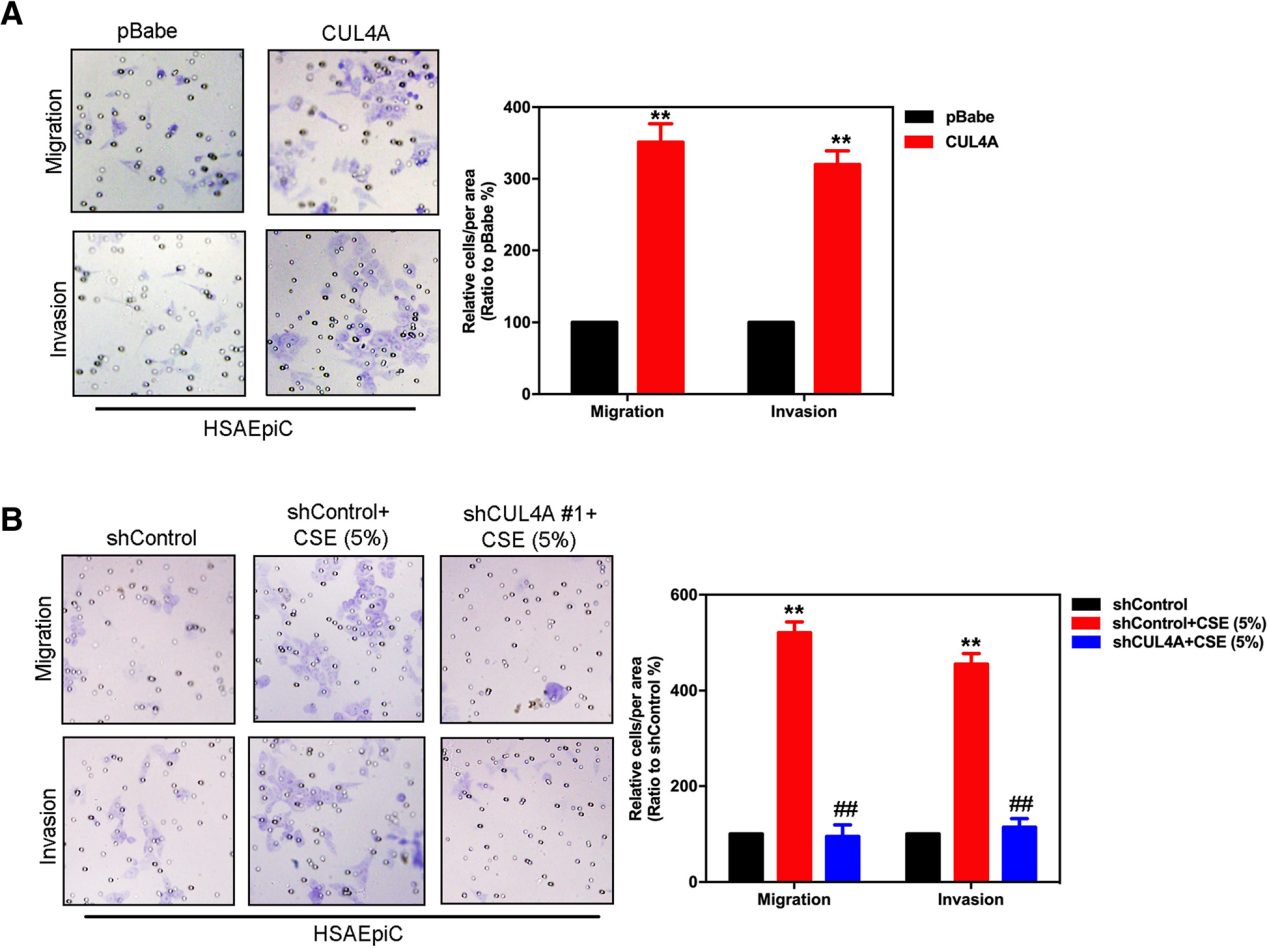
Effect of CUL4A on the invasion and migration ability of HSAEpiC cells. **a** CUL4A overexpression promotes invasion and migration of HSAEpic cells. **b** Knockdown of CUL4A inhibits CSE-induced invasion and migration of HSAEpic cells. **P < 0.01 compared to control group; ^##^*P* < 0.01 compared to shControl+CSE (5%) group. All results are from 3 independent experiments. Error bars indicate standard deviation

Numerous studies have confirmed that the occurrence of EMT can significantly affect the cell movement and invasion ability [9, 10, 19]. We further investigated whether CUL4A could regulate HSAEpiC cell movement and invasion. The results shown that overexpression of CUL4A significantly increased migration and invasion of HSAEpiC cells (Fig. 6a). We also found that knockdown CUL4A expression significantly reduces CSE-induced HSAEpiC cell migration and invasion (Fig. 6b). In summary, these results suggest that CUL4A plays an important role in CSE-induced HSAEpiC cell EMT.

### CUL4A up-regulates Slug expression in HSAEpiCs cells

**Fig. 7:**
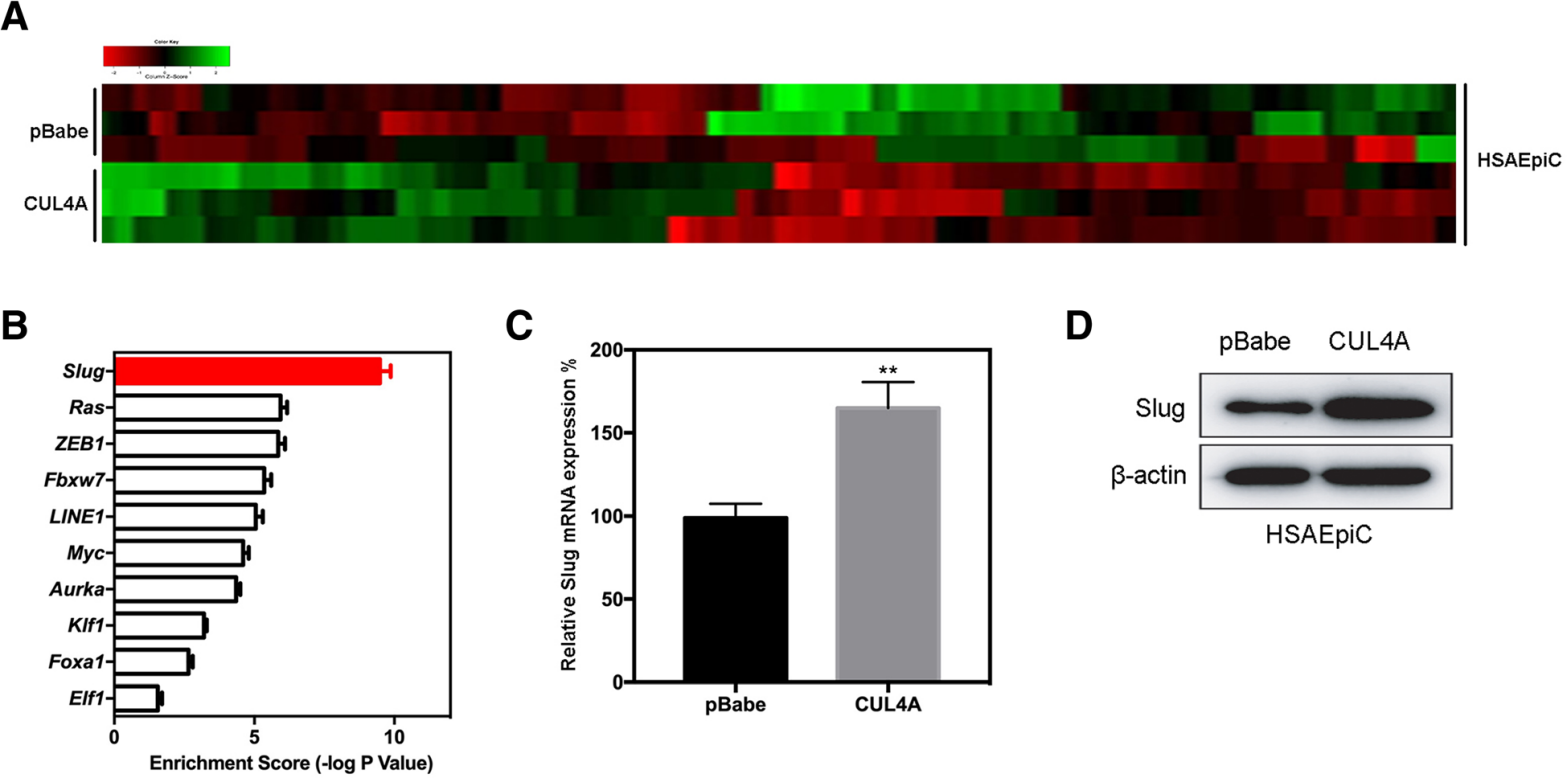
CUL4A up-regulated Slug expression in HSAEpiC cells. **a** A list of genes that were significantly differentially expressed after overexpression of CUL4A was identified by microarray analysis. **b** Gene enrichment analysis revealed that Slug was significantly enriched in CUL4A overexpressing cells. **c** qRT-PCR and **d** Western blot assessed the expression of Slug in HSAEpiC-CUL4A cells. **P < 0.01 compared to pBabe group. All results are from 3 independent experiments. Error bars indicate standard deviation

To better understand the mechanisms by which CUL4A is involved in the development and progression of COPD, we performed gene expression profiling of HSAEpiC-CUL4A and its control cells. Microarray analysis identified a list of genes that were significantly differentially expressed after CUL4A overexpression (Fig. 7a and Additional file 2: Table S1). In addition, gene enrichment analysis has shown that Slug is significantly enriched in CUL4A overexpressed cells (Fig. 7b), supporting the idea that CUL4A regulates proliferation, EMT, and invasion. This data also leads us to speculate that CUL4A may be performing these functions via Slug. To verify this, we first determine whether the Slug is a downstream target of CUL4A in HSAEpiC cell. Western blotting (Fig. 7d) and qRT-PCR (Fig. 7c) were used to further evaluate the expression of Slug in HSAEpiC-CUL4A cells. The results show that both mRNA and protein levels of Slug are significantly increased in HSAEpiC-CUL4A cells, indicating that CUL4A regulates Slug expression at the transcriptional level.

### CUL4A enriches H3K4me3 to the Slug gene promoter

**Fig. 8:**
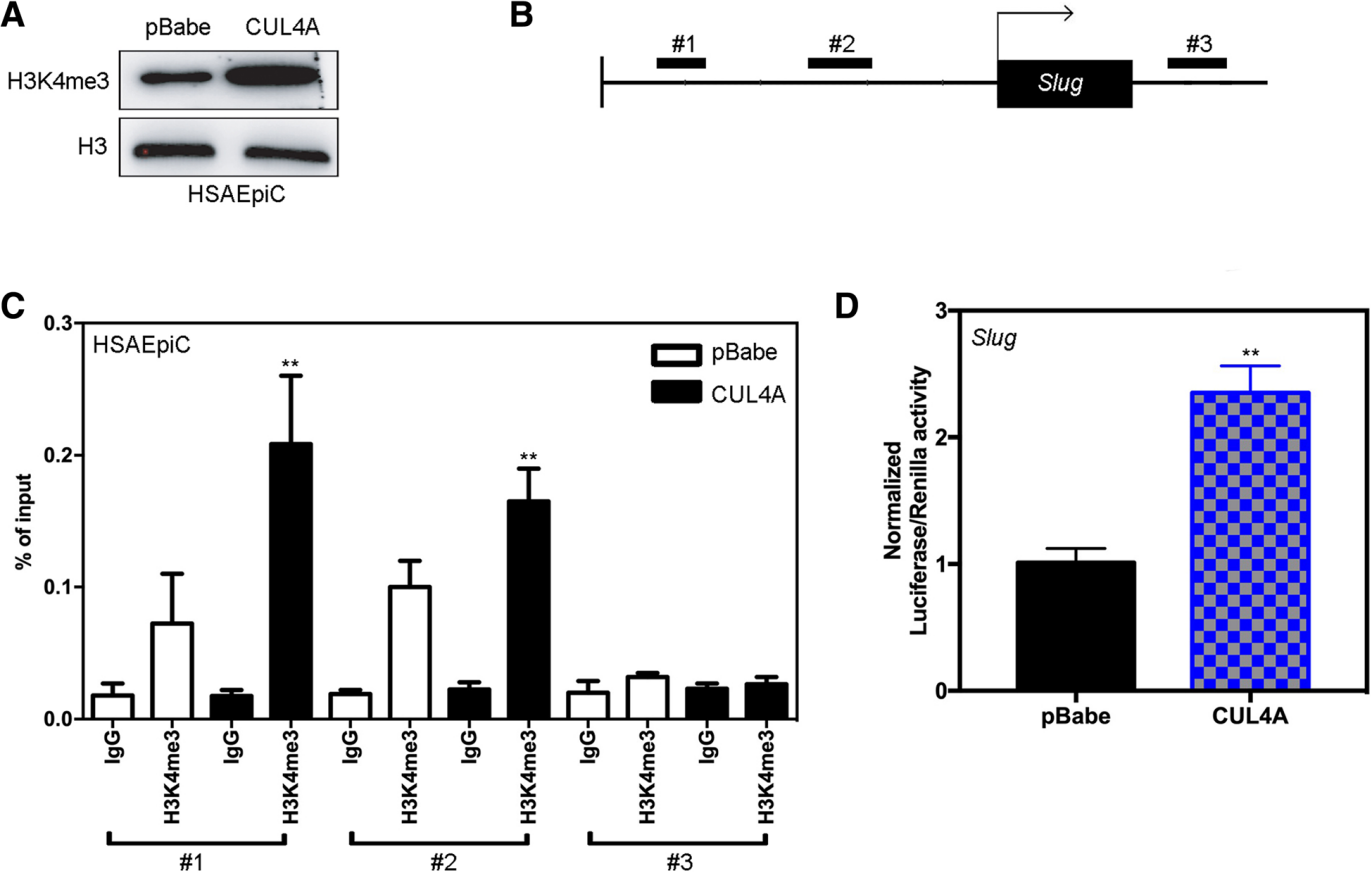
The expression of CUL4A is associated with an increase in the level of H3K4me3 on the Slug promoter. **a** Detection of histone modification after overexpression of CUL4A revealed that H3K4me3 was affected by overexpression of CUL4A. **b** Schematic presentation of three regions relative to the Slug transcriptional start site used as primers to test H3K14me3 occupied abundance. **c** ChIP-qPCR was performed to assess H3K4me3 occupancy to Slug transcriptional start site in HSAEpiC-CUL4A and its control cells. IgG was used as negative control. **d** Slug gene promoter luciferase assay was further confirmed. **P < 0.01 (in **c** and **d**) is based on Student’s t-test. Error bars indicate standard deviation

We then explore how CUL4A regulates Slug expression at the transcriptional level. Cullin-ring ligase complexes are often involved in chromatin regulation [21]. To determine whether CUL4A regulates specific histone modifications in HSAEpiC cells, we detect histone modification patterns after regulating CUL4A expression. We found that H3K4me3 is influenced by CUL4A expression, and CUL4A ectopic expression increases H3K4me3 (Fig. 8a). Since H3K4me3 is associated with active transcription, we examined whether CUL4A expression is related to H3K4me3 modification of Slug gene promoter in HSAEpiC cells. HSAEpiC-CUL4A and its control cells were detected by ChIP-qPCR. We found that the expression of CUL4A is related to the increased level of Slug promoter H3K4me3 in HSAEpiC-CUL4A cells (Fig. 8b and c), which is confirmed by Slug gene promoter luciferase assay (Fig. 8d). These results clearly indicate that CUL4A can induce transcriptional activation of Slug by regulating H3K4me3 and enriching H3K4me3 to Slug gene promoters.

### The highly positive correlation between CUL4A and Slug expression was found in the COPD patients lung tissues

**Fig. 9:**
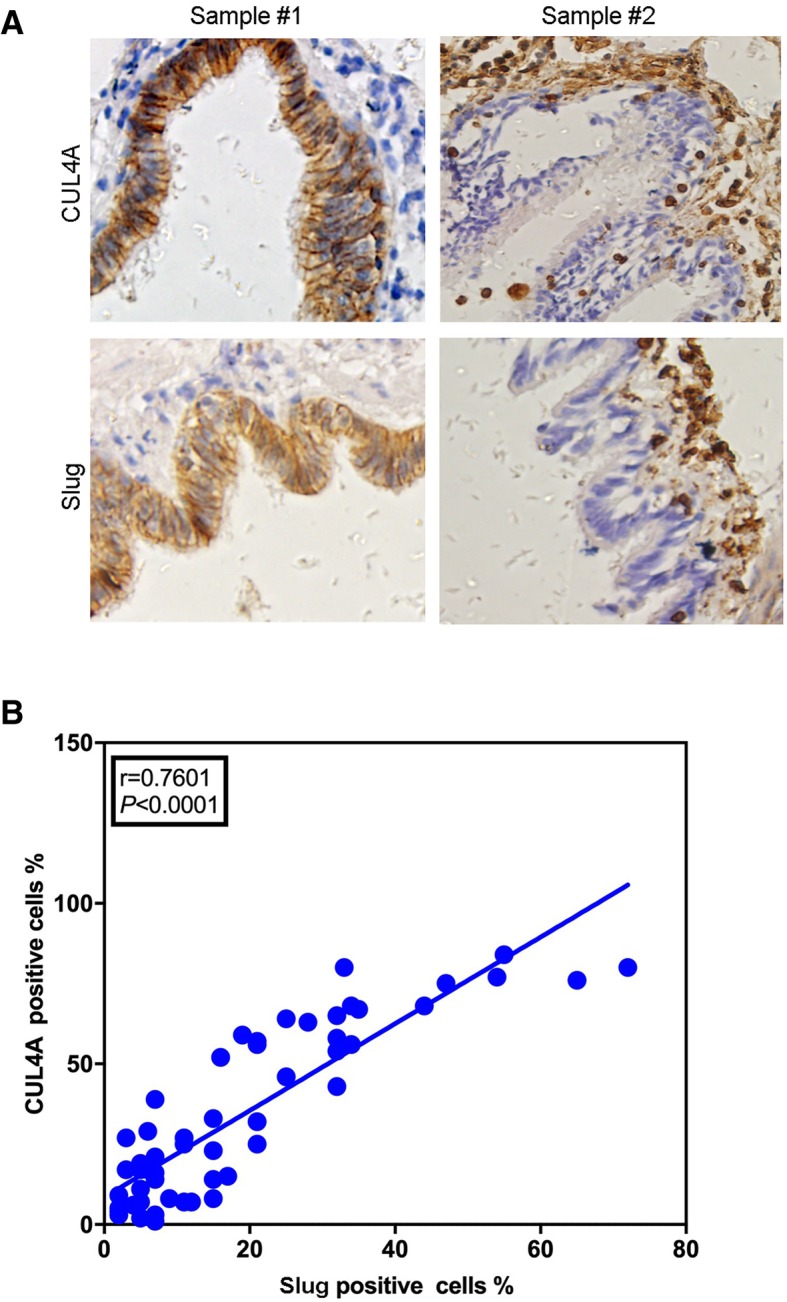
CUL4A is highly positively correlated with the expression of Slug in small airway epithelium of COPD. **a** The expression of CUL4A and Slug was analyzed by immunohistochemistry in small airway epithelium of COPD. **b** Correlations between the levels of CLU4A and the levels of Slug in small airway epithelium of COPD

To clarify the clinical correlation between CUL4A and Slug, we analyzed the expression of Slug in the same lung tissue by immunohistochemistry. CUL4A is highly positively correlated with the expression of Slug (Fig. 9a). Slug high expression is significantly correlated with CUL4A expression in COPD lung tissues (Fig. 9b). This result is consistent with the above in vitro analysis and further supports our study.

## Discussion

We have demonstrated for the first time that CUL4A is a mediator of small airway EMT in patients with COPD. Our results show that the expression of CUL4A in small airway epithelial are significantly higher in patients with COPD than in non-smokers and smokers with normal lung function, and there is a significant correlation between CUL4A and FEV_1_% of COPD. In vitro culture of HSAEpiC cells, CSE-induced EMT is associated with high CUL4A expression, and shRNA down-regulation of CUL4A inhibits CSE-induced EMT. Therefore, these evidences suggest that CUL4A is related to CSE-induced EMT. In addition, we also found that Slug may play an important role in CUL4A-mediated HSAEpiC cell EMT. These results collectively confirm that CUL4A is involved in small airway epithelial EMT in patients with COPD.

COPD is characterized by airflow limitation, which is not completely reversible [22]. The decrease of FEV_1_ is a characteristic of airflow limitation in this disease, which may be caused by small airway fibrosis [3]. Small airway fibrosis is the result of repair of damaged bronchiole epithelium after injury. Up to now, the exact mechanism of COPD small airway reconstruction remains unclear [23]. EMT refers to a series of phenotypic and molecular changes that occur not only at various stages of embryonic development but also as an important factor in fibrosis and cancer progression [7]. There are three types of EMT. Type I is associated with embryonic development and organogenesis, Type III is one of the important factors leading to the migration and invasion of cancer cells, and Type II EMT is closely related to inflammation. After inflammation or wound activation, it induces the production of fibroblasts and other related cells to play a role in wound repair. Airway remodeling is mainly related to repeated injury and repair of airway epithelial cells caused by airway inflammation. Although intratracheal EMT has been reported to be associated with the development of COPD, little is known about the underlying mechanisms of EMT in the stingy tubes of COPD [24].

We first assessed CUL4A expression in small airway epithelial cells based on a number of patients collected. In this study, we found that the expression of distal respiratory epithelial CUL4A increased in smokers and patients with the COPD compared with nonsmokers, especially in patients with chronic obstructive pulmonary disease. FEV_1_% was significantly negatively correlated with CUL4A expression. These results suggest that increased CUL4A levels may be associated with the occurrence of obstructive pulmonary disease. Tobacco smoke is the major etiological factor for the development of COPD and is a potent stimulus of DNA damage by oxidant injury. Tobacco smoke may affect the expression and activity of proteins associated with cell proliferation and cell apoptosis in both large airway epithelial and small airway epithelial cells. The limitations of this study are not staining of large airway epithelial cells with CUL4A from never smoker, former smoker and COPD. In the future study, we will focus on the CUL4A expression in large airway epithelial cells.

COPD is a heterogeneous disease associated with cigarette smoke exposure, which is believed to induce proteinase-mediated injury to the alveolar tissue and extracellular matrix, leading to emphysema. Due to the correlation between cigarette smoke and the pathogenesis of COPD, cigarette smoke has been widely used in vitro research. Consistent with other results, we cultured HSAEpiC cells with CSE. The results showed that after CSE treatment, the morphology of HSAEpic cells were fibroblast-like, the cells generally lost contact with each other, and the mRNA and protein expressions of the epithelial markers were down-regulated, and the mRNA and protein expression of mesenchymal markers increased. CUL4A mRNA and protein levels increased significantly after CSE exposure. To test whether CUL4A is related to CSE induced EMT, we inhibited CUL4A expression by shRNA, which inhibited CSE induced EMT in HSAEpiC cells. Overall, these results indicate that CSE-induced EMT is associated with CUL4A in HSAEpiC cells.

Recent evidence suggest that Slug participates in EMT in embryonic development and cancer metastasis by inhibiting the expression of their downstream target genes such as E-cadherin and occludin [25, 26]. In this study, we demonstrate that CUL4A induce transcriptional activation of Slug by regulating H3K4me3 and enriching H3K4me3 to Slug gene promoters, subsequently up-regulating the Slug expression in the HSAEpiC cells. Therefore, we support a model in which CSE-induced CUL4A expression activates cellular signaling pathways through multiple pathways that are complementary to the full-spectrum cell changes observed in induced EMT. The CUL4A-Slug chain may eventually promote EMT by regulating the expression of related genes.

## Conclusions

In summary, this study demonstrated that CUL4A is overexpressed in the distal airway of COPD and is related to the degree of airflow obstruction in COPD. We demonstrate that EMT-related molecular and phenotypic changes are due to CSE-induced CUL4A expression and activation of CUL4A-dependent cellular signals. We conclude that EMT may be induced in small airway epithelium in COPD patients through a mechanism involving activation of CUL4A signaling pathway.

## Additional files

Additional file 1: **Figure S1.** The proliferation of HSAEpiC cells were measured by BrdU-ELISA assay. A: The proliferation of HSAEpiC cells after CUL4A silencing was observed by BrdU-ELISA assay. B: The proliferation of HSAEpiC cells after CSE treated was observed by BrdU-ELISA assay. **P < 0.01 compared to shControl and CSE 0% group. All results are from 3 independent experiments. Error bars indicate standard deviation. (JPG 155 kb)

Additional file 2: **Table S1.** List of genes significantly differentially expressed after CUL4A overexpression in HSAEpiC (fold change ≥5). (DOCX 20 kb)

## Abbreviations

ChIP: Chromatin immunoprecipitation
COPD: Chronic obstructive pulmonary disease
CSE: Cigarette smoke extract
CUL4A: Cullin4A
EMT: Epithelial-mesenchymal transition
FEV1: Forced expiratory volume
HSAEpiC: Human small airway epithelial cells
