# Correction: The cullin4A is up-regulated in chronic obstructive pulmonary disease patient and contributes to epithelial-mesenchymal transition in small airway epithelium

**DOI:** 10.1186/s12931-023-02467-6

**Published:** 2023-07-14

**Authors:** Yidan Ren, Yi Zhang, Lixia Fan, Qinlian Jiao, Yunshan Wang, Qin Wang

**Affiliations:** 1 Department of Anesthesiology, Qilu Hospital, Shandong University, Jinan, China; 2 Department of Respiratory Medicine, Qilu Hospital, Shandong University, Jinan, China; 3 Department of Clinical Laboratory, The Second Hospital of Shandong University, Jinan, China; 4 International Biotechnology R&D Center, Shandong University School of Ocean, Weihai, China

**Correction: Respiratory Research (2019) 20:84** 10.1186/s12931-019-1048-4

Following the publication of this article [1], a reader drew to our attention an anomaly associated with the data shown in some figures. After careful scrutiny, we found that we somehow misused some images (Fig. 4A, E, F; Fig. 5A–C and Fig. 7D) in this article when constructing the final figure. This does not affect the interpretation of the experiments and the conclusions of the study. A corrected version of figure is presented here. We thank the reader of our article who drew this matter to our attention.


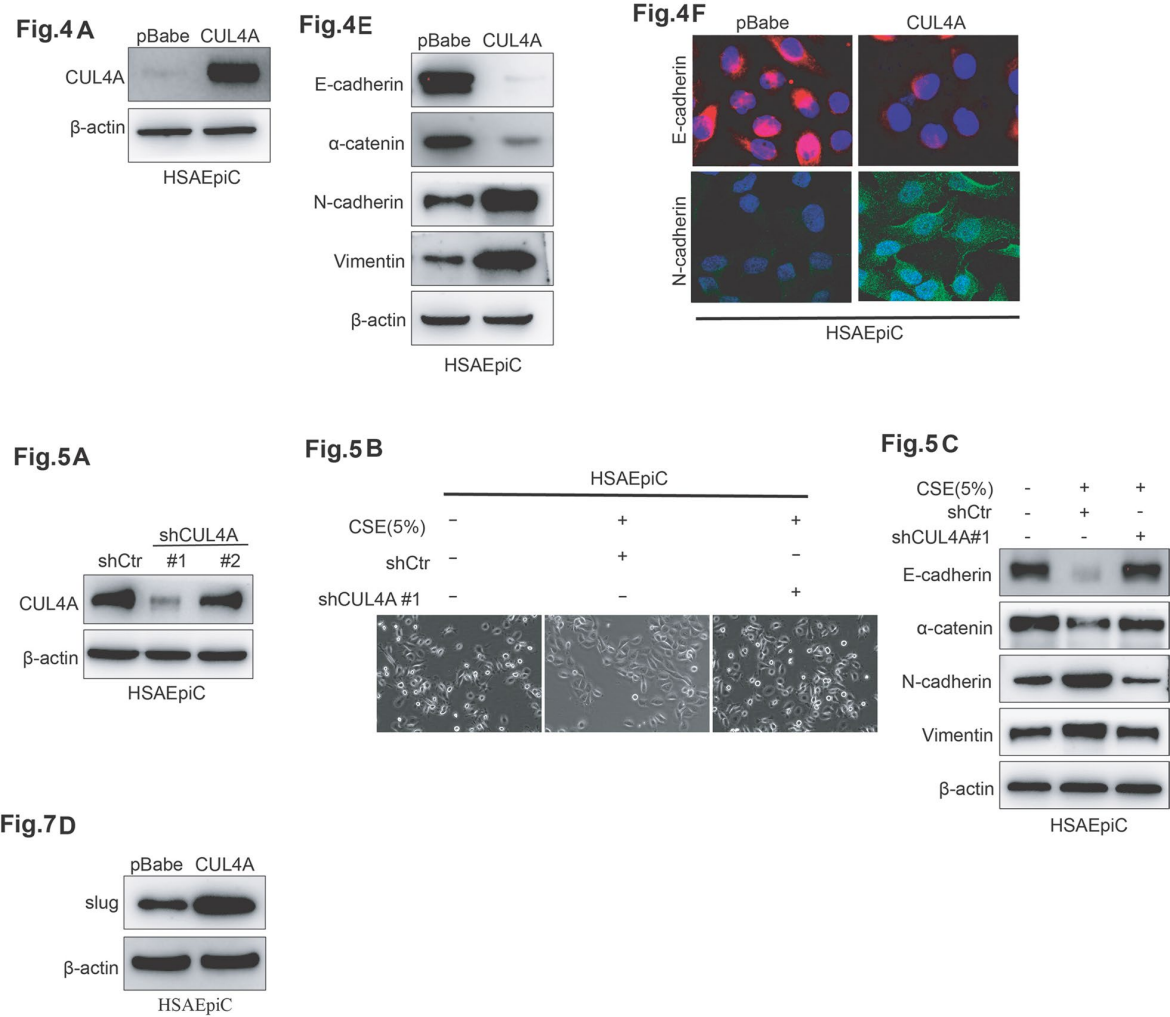


## References

[1] Ren Y, Zhang Y, Fan L, Jiao Q, Wang Y, Wang Q (2019). The cullin4A is up-regulated in chronic obstructive pulmonary disease patient and contributes to epithelial-mesenchymal transition in small airway epithelium. Respir Res.

