# Supplementary figures and images for: The cullin4A is up-regulated in chronic obstructive pulmonary disease patient and contributes to epithelial-mesenchymal transition in small airway epithelium
Source: Respir Res. 2019 May 6;20:84. doi: 10.1186/s12931-019-1048-4 (PMC6501375; doi:10.1186/s12931-019-1048-4)

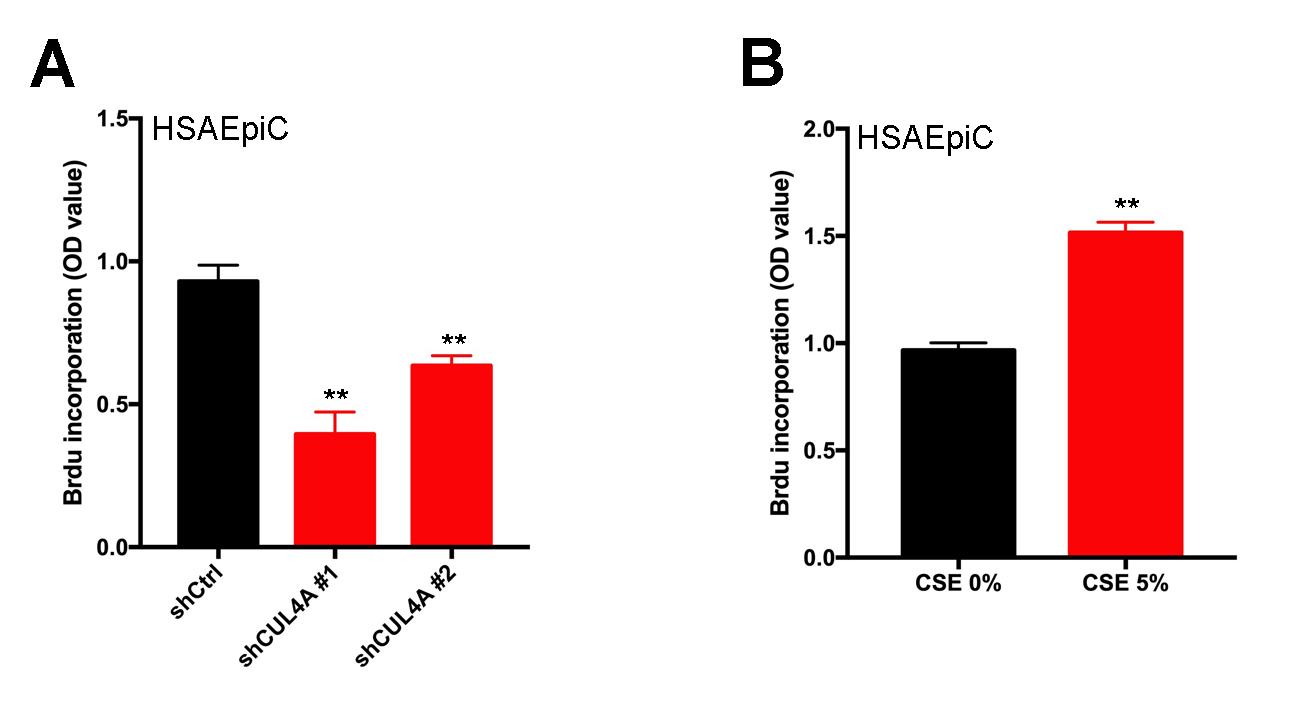

Supplement: Supplementary file 1 — Figure S1. The proliferation of HSAEpiC cells were measured by BrdU-ELISA assay. A: The proliferation of HSAEpiC cells after CUL4A silencing was observed by BrdU-ELISA assay. B: The proliferation of HSAEpiC cells after CSE treated was observed by BrdU-ELISA assay. **P < 0.01 compared to shControl and CSE 0% group. All results are from 3 independent experiments. Error bars indicate standard deviation. (JPG 155 kb) [file 12931_2019_1048_MOESM1_ESM.jpg]
