# Supplementary material for: The cullin4A is up-regulated in chronic obstructive pulmonary disease patient and contributes to epithelial-mesenchymal transition in small airway epithelium
Source: Respir Res. 2019 May 6;20:84. doi: 10.1186/s12931-019-1048-4 (PMC6501375; doi:10.1186/s12931-019-1048-4)
Supplement: Supplementary file 2 — Table S1. List of genes significantly differentially expressed after CUL4A overexpression in HSAEpiC (fold change ≥5). (DOCX 20 kb) [file 12931_2019_1048_MOESM2_ESM.docx]

**Supplemental Table 1.** **List of genes significantly differentially expressed after CUL4A overexpression in HSAEpiC (fold change ≥5)**

| **NCBI_GENE_ID** | **GENE_NAME** |
| --- | --- |
| 467 | Slug |
| 63976 | PRDM16 |
| 57787 | MARK4 |
| 23621 | BACE1 |
| 90231 | KIAA2013 |
| 93273 | LEMD1 |
| 2334 | AFF2 |
| 3561 | IL2RG |
| 55 | ACPP |
| 7066 | THPO |
| 399664 | RKHD1 |
| 83849 | SYT15 |
| 9627 | SNCAIP |
| 50484 | RRM2B |
| 337969 | KRTAP19-2 |
| N/A | N/A |
| 2100 | ESR2 |
| 8525 | DGKZ |
| 4793 | NFKBIB |
| 1813 | DRD2 |
| N/A | N/A |
| 387755 | LOC387755 |
| 387755 | LOC387755 |
| 7940 | LST1 |
| 29005 | PRO1073 |
| 4013 | LOH11CR2A |
| 10859 | LILRB1 |
| N/A | N/A |
| 11025 | LILRB3 |
| 1395 | CRHR2 |
| 8794 | TNFRSF10C |
| 26576 | STK23 |
| 259197 | NCR3 |
| 6006 | RHCE |
| 27130 | INVS |
| 9966 | TNFSF15 |
| 6560 | SLC12A4 |
| 5209 | PFKFB3 |
| 7439 | VMD2 |
| 7439 | VMD2 |
| 2847 | MCHR1 |
| 634 | CEACAM1 |
| 3690 | ITGB3 |
| 146225 | CMTM2 |
| 11123 | DSCR1L2 |
| 3486 | IGFBP3 |
| 379 | ARL4D |
| 78997 | GDAP1L1 |
| 5158 | PDE6B |
| 10983 | CCNI |
| 26470 | SEZ6L2 |
| 8614 | STC2 |
| 55603 | FAM46A |
| 51646 | YPEL5 |
| 79015 | MGC5566 |
| 2495 | FTH1 |
| 8519 | IFITM1 |
| 64400 | FTS |
| 92815 | HIST3H2A |
| 26136 | TES |
| 3162 | HMOX1 |
| 665 | BNIP3L |
| 27065 | D4S234E |
| 29923 | HIG2 |
| 573 | BAG1 |
| 3006 | HIST1H1C |
| 10608 | MXD4 |
| 55063 | ZCWPW1 |
| 6663 | SOX10 |
| 6273 | S100A2 |
| 3017 | HIST1H2BD |
| 51128 | SAR1B |
| 23645 | PPP1R15A |
| 8572 | PDLIM4 |
| 51741 | WWOX |
| 2909 | GRLF1 |
| 3705 | ITPK1 |
| 10468 | FST |
| 80759 | C6orf148 |
| 80772 | MGC10334 |
| 2353 | FOS |
| 55890 | GPRC5C |
| 84757 | MGC10814 |
| 4680 | CEACAM6 |
| 9976 | CLEC2B |
| 563 | AZGP1 |
| 4801 | NFYB |
| N/A | N/A |
| 344 | APOC2 |
| 9077 | DIRAS3 |
| 65251 | ZNF649 |
| 5319 | PLA2G1B |
| 6440 | SFTPC |
| 3493 | IGHA1 |
| 5452 | POU2F2 |
| 3725 | JUN |
| 57379 | AICDA |
| 3500 | IGHG1 |
| 7695 | ZNF136 |
| 10110 | SGK2 |
| 9022 | CLIC3 |
| 8674 | VAMP4 |
| 4502 | MT2A |
| 84839 | RAXL1 |
| 84841 | MGC15634 |
| 84332 | MGC16186 |
| 11132 | CAPN10 |
| 1054 | CEBPG |
| 23504 | RIMBP2 |
| 84970 | C1orf94 |
| 23493 | HEY2 |
| N/A | N/A |
| N/A | N/A |
| 84989 | MGC14425 |
| 6662 | SOX9 |
| 952 | CD38 |
| N/A | N/A |
| 10101 | NUBP2 |
| 246329 | STAC3 |
| 885 | CCK |
| 948 | CD36 |
| 29923 | HIG2 |
| N/A | N/A |
| 30001 | ERO1L |
| 92342 | C1orf156 |
| 2947 | GSTM3 |
| 9518 | GDF15 |
| 90423 | ATP6V1E2 |
| 163154 | MGC24975 |
| 90050 | C14orf152 |
| 8828 | NRP2 |
| 3303 | HSPA1A |
| 11135 | CDC42EP1 |
| 90701 | SEC11L3 |
| 116442 | RAB39B |
| 64081 | MAWBP |
| 9709 | HERPUD1 |
| 2623 | GATA1 |
| 23302 | KIAA0523 |
| 53917 | RAB24 |
| 80199 | FLJ22688 |
| 115708 | C14orf172 |
| 1475 | CSTA |
| 6710 | SPTB |
| 25789 | C19orf4 |
| 929 | CD14 |
| 1385 | CREB1 |
| 9071 | CLDN10 |
| 8365 | HIST1H4H |
| 1448 | CSN3 |
| 894 | CCND2 |
| 84923 | FAM104A |
| 57348 | TTYH1 |
| 55172 | C14orf104 |
| 3108 | HLA-DMA |
| 3488 | IGFBP5 |
| 10013 | HDAC6 |
| 54814 | QPCTL |
| 10681 | GNB5 |
| 1647 | GADD45A |
| 4610 | MYCL1 |
| 5080 | PAX6 |
| N/A | N/A |
| 54584 | GNB1L |
| 407 | ARR3 |
| 2632 | GBE1 |
| 6548 | SLC9A1 |
| 51573 | MIR16 |
| 3538 | IGLC2 |
| 2173 | FABP7 |
| 23452 | ANGPTL2 |
| 9823 | ARMCX2 |
| 55002 | TMCO3 |
| 55512 | SMPD3 |
| 149603 | RNF187 |
| N/A | N/A |
| N/A | N/A |
| 3340 | NDST1 |
| 4601 | MXI1 |
| 51085 | MLXIPL |
| 30001 | ERO1L |
| 3111 | HLA-DOA |
| 25849 | DKFZP564O0823 |
| 83667 | SESN2 |
| 3772 | KCNJ15 |
| 1317 | SLC31A1 |
| 4916 | NTRK3 |
| 26 | ABP1 |
| 90993 | CREB3L1 |
| 251 | ALPPL2 |
| 1995 | ELAVL3 |
| 114780 | PKD1L2 |
| 3500 | IGHG1 |
| 23612 | PHLDA3 |
| 6649 | SOD3 |
| 11126 | CD160 |
| 634 | CEACAM1 |
| 130951 | LOC130951 |
| 3500 | IGHG1 |
| 3664 | IRF6 |
| 8535 | CBX4 |
| 7439 | VMD2 |
| 64147 | KIF9 |
| 3911 | LAMA5 |
| 3429 | IFI27 |
| 8530 | CST7 |
| 10142 | AKAP9 |
| 196872 | MGC23270 |
| 3662 | IRF4 |
| 81706 | PPP1R14C |
| 4057 | LTF |
| 4057 | LTF |
| N/A | N/A |
| 10964 | IFI44L |
| 55670 | PEX26 |
| 8294 | HIST1H4I |
| 3493 | IGHA1 |
| N/A | N/A |
| 256691 | MAMDC2 |
| 54988 | FLJ20581 |
| 2972 | BRF1 |
| 3078 | CFHR1 |
| 219928 | MRGPRF |
| 22861 | NALP1 |
| 54923 | LIME1 |
| 7429 | VIL1 |
| 8334 | HIST1H2AC |
| 6696 | SPP1 |
| 79983 | POF1B |
| 27033 | SLUG |
| 6928 | TCF2 |
| 9476 | NAPSA |
| 10786 | SLC17A3 |
| 91543 | RSAD2 |
| 8740 | TNFSF14 |
| 151871 | DPPA2 |
| 8153 | RND2 |
| 2869 | GRK5 |
| 130576 | LOC130576 |
| 4143 | MAT1A |
| 6947 | TCN1 |
| 805 | CALM2 |
| 84951 | TNS4 |
| 63940 | GPSM3 |
| 3500 | IGHG1 |
| 28815 | IGLV2-14 |
| 22927 | HABP4 |
| 6919 | TCEA2 |
| 3500 | IGHG1 |
| 79794 | C12orf49 |
| 84466 | MEGF10 |
